# ‘Who’s a good boy?!’ Dogs prefer naturalistic dog-directed speech

**DOI:** 10.1007/s10071-018-1172-4

**Published:** 2018-03-02

**Authors:** Alex Benjamin, Katie Slocombe

**Affiliations:** Department of Psychology, The University of York, York, UK

**Keywords:** Dog-directed speech, Human–dog communication, Infant-directed speech, Dog cognition, Affiliative behaviour, Dog attention

## Abstract

Infant-directed speech (IDS) is a special speech register thought to aid language acquisition and improve affiliation in human infants. Although IDS shares some of its properties with dog-directed speech (DDS), it is unclear whether the production of DDS is functional, or simply an overgeneralisation of IDS within Western cultures. One recent study found that, while puppies attended more to a script read with DDS compared with adult-directed speech (ADS), adult dogs displayed no preference. In contrast, using naturalistic speech and a more ecologically valid set-up, we found that adult dogs attended to and showed more affiliative behaviour towards a speaker of DDS than of ADS. To explore whether this preference for DDS was modulated by the dog-specific words typically used in DDS, the acoustic features (prosody) of DDS or a combination of the two, we conducted a second experiment. Here the stimuli from experiment 1 were produced with reversed prosody, meaning the prosody and content of ADS and DDS were mismatched. The results revealed no significant effect of speech type, or content, suggesting that it is maybe the combination of the acoustic properties *and* the dog-related content of DDS that modulates the preference shown for naturalistic DDS. Overall, the results of this study suggest that naturalistic DDS, comprising of both dog-directed prosody and dog-relevant content words, improves dogs’ attention and may strengthen the affiliative bond between humans and their pets.

**Electronic supplementary material:**

The online version of this article (10.1007/s10071-018-1172-4) contains supplementary material, which is available to authorized users.

## Introduction

When talking to an infant, adults use a special speech register characterised by elevated fundamental frequency (pitch), exaggerated intonation contours and high affect (Burnham et al. 2002). This phenomenon is evident across languages including English, Russian, Swedish and Japanese (Kuhl et al. 1997; Andruski et al. 1999). It is thought that infant-directed speech (IDS) facilitates infants’ linguistic development by amplifying the phonetic characteristics of native language vowels (Kuhl et al. 1997), allows infants’ to select appropriate social partners (Schachner and Hannon 2011) and increases social bonding between infant and caregiver (Kaplan et al. 1995).

In the same way that IDS is produced automatically when talking to infants, humans in Western cultures also produce a special speech register when talking to their pets. This pet-directed speech (PDS) shares some of the acoustic features of IDS including elevated pitch and exaggerated affect compared to adult-directed speech (ADS) (Burnham et al. 1998). It is possible that pitch is elevated in IDS and PDS in order to attract the listener’s attention, while affect is elevated to meet listener’s emotional needs, possibly motivating affiliative interaction with the speaker. One crucial feature not shared between IDS and PDS and only found in IDS is the hyperarticulation of vowels (Burnham et al. 1998). Hyperarticulation of vowels may be the aspect of IDS that assists spoken language acquisition (Kuhl et al. 1997) and the speaker’s hyperarticulation may be mediated by the perceived linguistic capacity of the receiver; evidence that supports this view is provided by a study that compared speech produced to dogs, parrots and infants. Speakers seem to hyperarticulate their vowels most with prelinguistic human infants, followed by parrots, with little evidence of this when addressing dogs, who in contrast to parrots have no ability to produce speech (Xu et al. 2013).

It is evident that speakers are sensitive to their audience in terms of acoustic preference, emotional needs and linguistic potential; however, in order to understand the function of special speech registers, it is crucial to understand how they affect the receiver. Human infants show a preference for IDS from a very early age (Kaplan et al. 1995), with Cooper and Aslin (1990) finding preferences for IDS over ADS in 2-day-old infants. Werker and McLeod (1989) measured affective responsiveness to ADS and IDS in 4–5- and 7–9-month-old infants. Two trained raters judged the affective responsiveness of infants, comprising of how much they thought the infant was trying to interact with the speaker, how interested they appeared and the valence of the infant’s emotional state. They found that infants of both age groups showed greater affective responsiveness to IDS than to ADS. They also found that when presented with video recordings of infants listening to speech, unfamiliar observers rated the infants more ‘appealing’ when the infants were listening to IDS than when they were listening to ADS. This indicates that the use of IDS may facilitate the development of an emotional bond between adults and infants. In contrast to IDS, there has been very little research into the effect of PDS on receivers, meaning that it is currently unclear whether PDS is a non-functional overgeneralisation of IDS in Western cultures where pets often have the status of infants or whether it functions to gain pets’ attention and strengthen the affiliative bond between humans and their pets.

Ben-Aderet et al. (2017) were the first to investigate both the production of dog-directed speech (DDS) and the behavioural response to DDS in puppies, adult dogs and older dogs. Acoustic analysis of DDS confirmed previous descriptions of the acoustic structure of this speech register, where DDS was higher in pitch, with more pitch variation over time, and higher harmonicity than ADS. They also showed that human adults produced DDS to dogs of all ages. Crucially, Ben-Aderet et al. (2017) then conducted playback experiments using the DDS and ADS recorded in the first part of the study to test dog responses to these types of speech. Stimuli consisted of repetitions of the phrase *‘Hi! Hello cutie! Who’s a good boy? Come here! Good Boy! Yes! Come here sweetie pie! What a good boy!*’ in dog- and adult-directed prosody. Speech was played from a loudspeaker in the corner of the room, with no human near the source of the sound and various measures of dogs’ attention to and approach of the loudspeaker were combined into a composite behavioural response measure. They found that puppies showed a higher behavioural response to DDS than for ADS, but this preference decreased as a function of age. The authors conclude that puppies are highly reactive to DDS and that pitch is a key feature in modulating this preference, but that adult dogs do not react differentially to DDS and ADS. They argue that DDS may have a functional value in puppies, but not adult dogs, and therefore, the use of DDS with adult dogs may simply be a ‘spontaneous attempt to facilitate interactions with non-verbal listeners’ (Ben-Aderet et al. 2017, p. 1). It is, however, possible that alternative explanations of the null result with adult dogs exist. As Ben-Aderet et al. discuss, adult dogs may need additional cues (e.g. gestures) to respond to unfamiliar speakers. If DDS functions to facilitate social communication and interaction, it may only be relevant to attend to it when it comes from a human that can be attended to and socialised with. It is possible that if no human experimenter is present, adult dogs realise that there is no social benefit to reacting preferentially to any speech. Puppies, with little experience of the world, may not recognise this and therefore still responded to DDS in the absence of a feasible producer. While it is clear that puppies are more reactive to the prosody of DDS than adult dogs, further testing with a human speaker present during stimulus presentation is required in order to rigorously test whether adult dogs really are insensitive to DDS. We therefore aimed to test the possible function of DDS with adult dogs in a more ecologically valid setting where attention and affiliation towards the individuals who produced DDS could be directly measured. Dogs were presented with two experimenters with audio speakers on their laps that played naturalistic DDS or ADS (differing in both prosody and content), and we measured the dogs’ attention to each individual during speech and then proximity to the experimenters once dogs were given the opportunity to approach them after the speech finished. We predicted that if DDS is functional for adult dogs, in experiment 1 they should attend more to DDS than ADS, and when given the opportunity to approach the experimenters, they should choose to spend more time in proximity to the individual who produced DDS. We then ran a second experiment to investigate whether content or prosody was driving any preferences for naturalistic DDS. Here we presented content-mismatched stimuli (e.g. adult content with dog prosody and vice versa) and predicted that if the content of naturalistic DDS was driving preferences, dogs should attend to and spend more time near the individual producing dog-relevant content. If, on the other hand, the prosody of DDS was driving preferences, as was the case for the puppies studied by Ben-Aderet et al. (2017), dogs should attend to and spend more time near the individual producing dog-directed prosody. Finally if preferences for naturalistic DDS are driven by both content and prosody, or result from the combination of dog-relevant content and DDS prosody, we expect to find no significant preference for either of the mismatched stimuli.

## Experiment 1

As we were interested in naturalistic dog- and adult-directed speech, the stimuli used in this experiment varied in both content and prosody. The stimuli were ‘matched’ in prosody and content such that DDS consisted of dog-relevant content and dog-directed prosody, and ADS consisted of adult-relevant content and adult-directed prosody.

### Methods

#### Study site and participants

Dogs were recruited from Redhouse Boarding Kennels, York, with permission from the kennel owner. In experiment 1, 37 dogs took part (17 females and 20 males; mean age 6 years ± 3.86) in this study between January and May 2014. See supplementary material for more detailed age, gender and breed information (Table S1). Where dogs have been removed from various parts of the analysis due to interruptions, equipment failures or safety reasons, the details and N for each analysis are given.

#### Stimuli

Stimuli were recorded as uncompressed WAV files using a Marantz PMD661 solid-state recorder from the two human female experimenters (aged 20–21). The recordings from experimenter A were always presented through experimenter A’s speaker (and the same for experimenter B), ensuring congruency of speech with physical characteristics. Although only presenting speech from the experimenters meant that multiple dogs heard the same recordings, it ensured that the stimuli were congruous with the physical characteristics of the experimenters (age, gender, height), thus maximising ecological validity and removing the possibility of looking time measures being affected by incongruity of the stimuli. DDS was chosen from a sample of recorded naturalistic interactions with a friendly dog (irish setter). ADS was chosen from a sample of naturalistic adult–adult interactions that occurred between the experimenters (see supplementary material for transcripts).

**Fig. 1:**
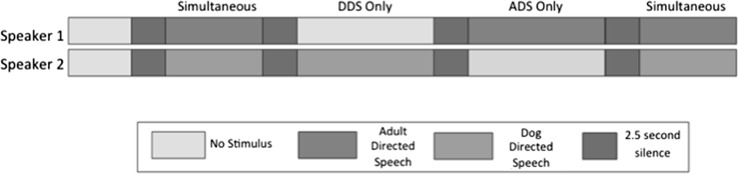
A diagram illustrating the stimulus timeline. ADS only and DDS only segments were counterbalanced such that half the dogs heard ADS only first and half heard DDS only first. Each track was played simultaneously (DDS from one speaker, ADS from another speaker) from an iPod paired with an Anchor speaker. The same 10-s segment was used in simultaneous 1 and 2 for each speaker, though these segments differed from the 15-s segments in ADS and DDS only phases

Two different segments of DDS and ADS for each experimenter were selected from the continuous speech recordings (one 10-s segment and one 15-s segment). The amplitude of the speech in each segment was modified using Raven Pro (version 1.4), so that the mean RMS amplitude of each segment was equalised at approximately 3000. For each trial, the DDS track of one experimenter was paired with the ADS track of another. Figure 1 illustrates the stimulus timeline.

#### Design

This experiment used a within-subject design, where all dogs heard both DDS and ADS. All dogs heard simultaneous speech first, followed by DDS only and ADS only. The order of DDS and ADS only segments was counterbalanced across trials. Simultaneous was played again at the end, to eliminate the possibility that dogs would approach the individual who spoke last. We also counterbalanced the identity of the DDS speaker (experimenter 1 or 2) and the location from which DDS was played (left/right) across trials.

#### Procedure

**Fig. 2:**
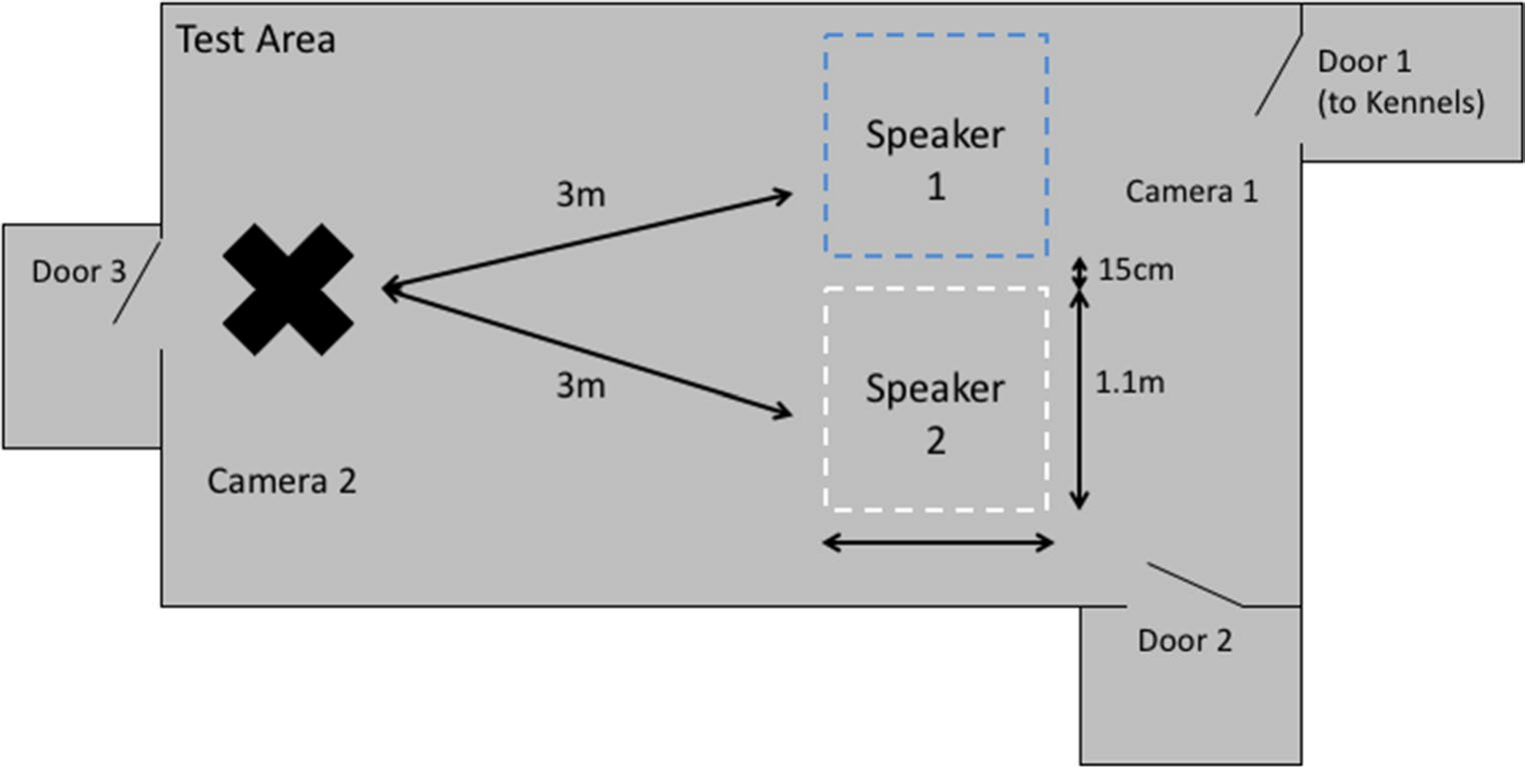
Diagram of experimental set-up at Redhouse Boarding Kennels in York. Position of dog marked with a cross. Cameras were positioned behind and to the right of the dog, and behind the speakers. Doors to other areas are marked. Dotted lines represent edges of areas in which proximity to speaker was recorded. Experimenters with speakers on their laps were seated on chairs in the centre of each area

Equipment was set up as illustrated in Fig. 2. The speakers were equalised to 70 dB at 1 m away with white noise using a sound pressure meter, to ensure that that speech broadcast from each speaker would be equal in volume. Experimenters 1 and 2 then left the room via door 2. The third experimenter (handler) retrieved the dog from its kennel and entered the experimental room through door 1. The dog was allowed to explore the experimental room for 1 min (to habituate to the environment in order to reduce distraction during the trial), before being put back on a lead and taken into a waiting room via door 3. Experimenters 1 and 2 entered through door 2 and sat in the chairs. The handler entered with the dog. Once the dog was in position, the stimulus was played.

For the duration of the stimulus, the experimenters sat still to ensure the dogs were not exposed to any body language cues. The experimenters did not attempt to move their mouths simulating the speech. Instead, the experimenters placed one hand covering their mouths so that the dog could not see their lips. They also maintained neutral expressions with eyes directed towards the dog to ensure the dog did not receive differential facial cues from the experimenters.

While the stimulus played, the dog was kept on a short lead to ensure it remained within camera visibility, while still allowing the dog to move around within 1 m of the handler. The handler did not interact with the dog and looked at the ground throughout. At the end of the stimulus phase, the lead was removed and the dog was allowed to explore freely for 1 min and approach experimenters 1 and 2 if they wished. The dog received no interaction from any experimenter.

#### Video coding

Video recordings of each session were analysed, and during the stimulus presentation, time spent looking towards DDS and ADS was recorded as measured by head direction. During the 1-min off-lead period following the stimulus presentation, time spent in proximity to DDS and ADS speakers was recorded, as measured by the position of the dog’s head in the 1.1 m^2^ area surrounding the speaker (see Fig. 2).

The period after the dog entered the room, but before the stimulus began was used as a control period (mean duration 4.56 ± 2.14 s). Looking times during this phase were recorded in order to establish whether the dog displayed any preference for one experimenter in particular, or one location (left or right) that may have influenced looking times in the experiment.

#### Interobserver reliability

The primary observer (AB) coded 100% of videos. For experiment 1, two trained observers each coded 30% of videos (*N* = 24/36 trials total) and measured looking time at each speaker in each section of the stimulus (control silence, simultaneous 1, DDS only, ADS only, simultaneous 2; *N* = 10 measurements) and time in proximity to each speaker in the minute post-stimulus presentation (*N* = 2 measurements). The primary coder had high agreement with the two secondary coders, and there was also high agreement between the two secondary coders across all measurements (Spearman’s *R* > 0.90, *p* < 0.001 for all comparisons), indicating the videos had been coded reliably.

A third observer, who was blind to the hypotheses of the experiment, also coded 22% of the videos (*N* = 8/36 trials total) with the sound turned off so that they were unaware which speech type was heard by the dog. There was high agreement with the primary coder for looking time (*R* = 0.86, *p* < 0.001) and for proximity preference (*R* = 0.96, *p* < 0.001).

#### Statistical analysis

All data were analysed using IBM SPSS (version 24) with the significance level set at *p* < .050. Attentive and affiliative preference was evaluated using mixed ANOVAs with the fixed within-subject factor speech prosody (DDS/ADS), between-subject factors DDS identity (experimenter 1/experimenter 2) and DDS location (right/left). A single mixed ANOVA was conducted on the proximity to speakers in the minute post-stimulus presentation. For looking time, after the ANOVA on the total looking time had been completed (Table 1), separate ANOVAs were then run for each section of the stimulus (simultaneous; ASD only; DDS only). We applied a more conservative Bonferroni-corrected alpha level to the separate section analyses (*p* = 0.01) to correct for family-wise error that might have arisen from running multiple tests on the same data set. Finally, we ran an ANOVA with between-subject factors DDS identity (experimenter 1/experimenter 2) and DDS location (right/left) on proportion of looking times in the control period. All assumptions of these parametric tests were tested and met.

**Table 1:** Results of a between-subject ANOVA (*df* = 1, 29) on looking proportions in the control period and a mixed ANOVA (*df* = 1, 29) comparing main effects and interactions for looking times towards content-matched DDS and ADS

	Within-subject effects F(*p*)				Between-subject effects F(*p*)		
	Speech type	Speech type *identity	Speech type * location	Speech type * identity * location	Identity	Location	Identity * location
Control silence					0.38 (.543)	0.59 (.448)	0.85 (.364)
Total looking	**40.51 (**< .001)***	0.15 (.704)	1.61 (.215)	0.24 (.627)	0.20 (.656)	1.37 (.251)	0.43 (.517)

Bold value denotes a significant finding

Significant results are marked, where *** denotes *p* < 0.005

### Results

#### Looking preference

**Fig. 3:**
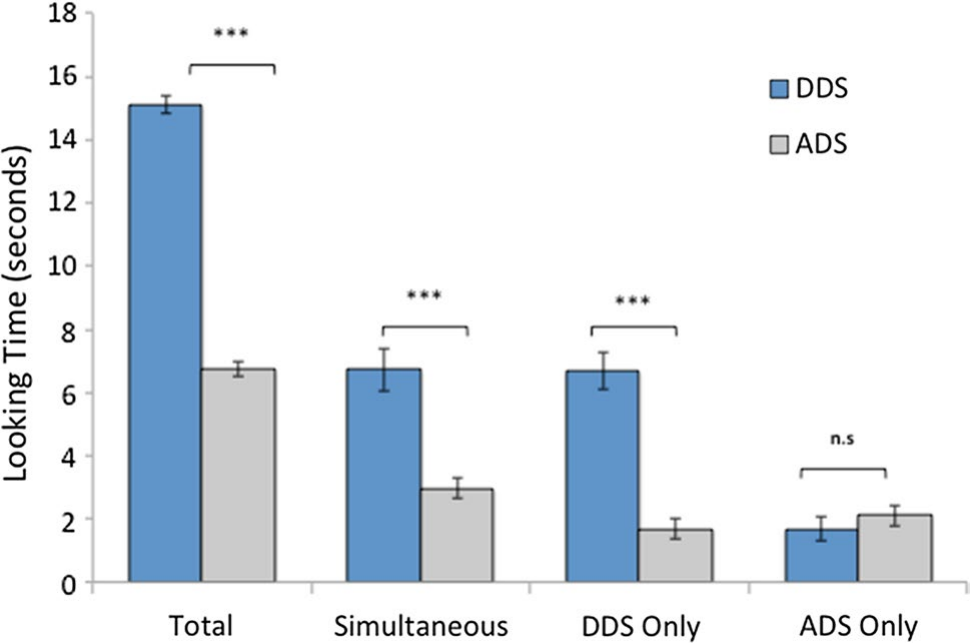
Time spent looking towards content-matched DDS and ADS where error bars represent 1 standard error of the mean. ***refers to significant differences (*p* < 0.005) and n.s denotes non-significant comparisons as revealed by mixed ANOVAs (total: Table 1; other time segments Table S3)

For this analysis, four subjects were removed due to equipment failure (*N* = 33). During control silence, there was no significant main effect of Identity or Location, indicating that dogs did not display any preference for one particular experimenter or speaker location (Table 1). Dogs displayed a significant preference for DDS across the whole trial (Fig. 3; Table 1) and during each phase that contained DDS (Fig. 3; Table S3). Dogs tended to look more towards ADS when this was the only stimulus available; however, this preference was non-significant (Fig. 3). No significant interactions with speaker identity or location were found for total time (Table 1) or separate segments of the stimuli (simultaneous, DDS only, ADS only) (Supplementary Material: Table S3).

#### Proximity preference

**Fig. 4:**
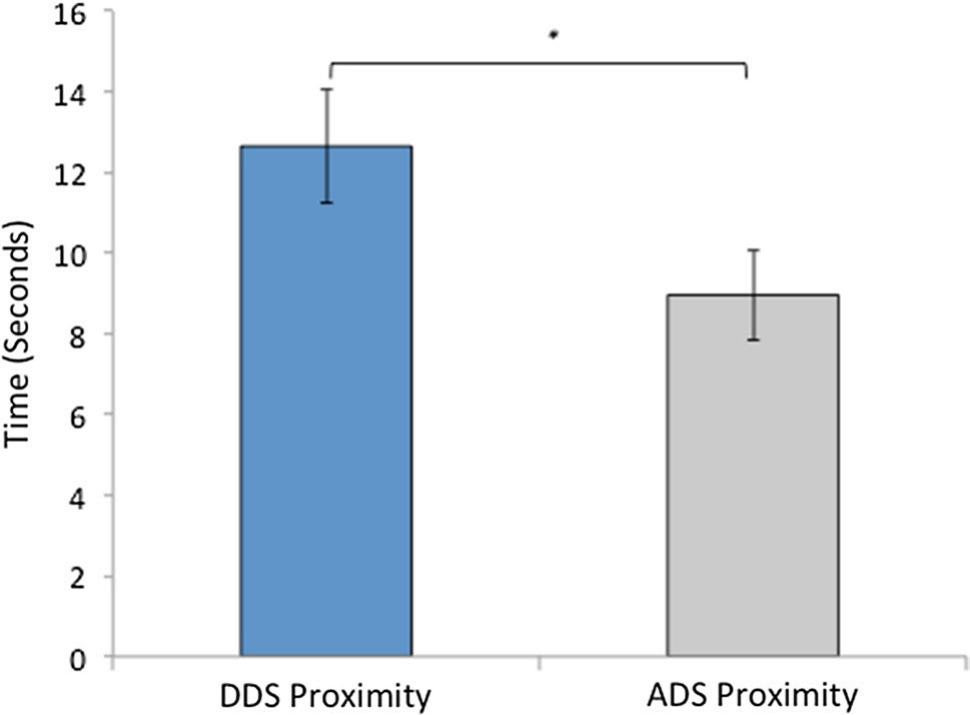
A graph to show the mean time spent in proximity to each experimenter (seconds), in the minute after the speech stimuli ended, when the dogs heard content-matched DDS and ADS. Error bars represent one standard error of the mean. (*) denotes a significant main effect of speech type (*p* < 0.050) based on the results of ANOVA presented in Table 2

For this analysis, three dogs were removed from the data set due to equipment failure or because the dog had to be kept on a lead, resulting in an *N* = 34. A mixed ANOVA revealed that after hearing content-matched stimuli, dogs spent significantly more time in close proximity to the DDS speaker than the ADS speaker (F (1, 30) = 5.54, *p* = 0.025; Fig. 4). No significant interactions with location or speaker identity were found (Table 2).

**Table 2:** Results of a mixed ANOVA with degrees of freedom (1, 30) comparing the time spent near DDS and ADS speakers for content-matched speech

	Within-subject effects *F*(*p*)				Between-subject effects *F*(*p*)		
	Speech type	Speech type *identity	Speech type * location	Speech type * identity * location	Identity	Location	Identity * location
Proximity preference	5.54 (.025)*	1.64 (.210)	0.29 (.592)	0.05 (.833)	1.13 (.552)	0.36 (.552)	0.62 (.438)

Bold value denotes a significant finding

Significant results indicated, where * denotes *p* < 0.050

### Discussion

This experiment showed that dogs display a behavioural preference for naturalistic DDS (matched in prosody and content) compared with ADS when presented in the presence of an associated human. Dogs, on average, spent more time looking towards a speaker of DDS compared with a speaker of ADS in all segments of the stimulus containing DDS and across the trial as a whole. We also found that when given the subsequent opportunity to interact with the speakers, dogs chose to spend more time in proximity with the DDS speaker, than the ADS speaker. Although the absolute differences in looking and proximity time were small and therefore their functional relevance may be questioned, we feel the substantial effect sizes obtained and the convergence of results across our behavioural measures indicates we have detected functionally relevant differences in behaviour. Overall, our results support the hypothesis that dogs display attentive and affiliative preferences for naturalistic DDS over ADS.

The results from the control period show no significant preference for a specific location, or speaker identity, indicating that the dogs had no a priori preference for looking at one experimenter or location. In line with this, no significant main effects of location or speaker identity, or interactions of identity, location and speech type were found.

Although our results show a robust preference for naturalistic DDS over ADS, as the stimuli in this experiment differed in both content and prosody, it is not possible to determine whether this effect is driven by dog-directed prosody or content, as these factors did not vary independently. Therefore, although this experiment clearly shows that dogs discriminate between and show a behavioural preference for naturalistic DDS over ADS, further investigation is required to determine the extent to which prosody and content are driving this preference.

## Experiment 2

Experiment 2 was designed in order to examine whether content alone or prosody alone was sufficient for driving the preference found in experiment 1. In experiment 2, the content from experiment 1 was reproduced but with reversed prosody such that the dog-related content was spoken with the prosody of ADS and vice versa. For simplicity, in all cases, DDS refers to stimuli with dog-directed prosody (with either dog- or adult-related content) and ADS refers to stimuli with adult-directed prosody (with either adult- or dog-related content). In experiment 2, we presented dogs with content-mismatched DDS (dog-directed prosody with adult-related content) and content-mismatched ADS (adult-directed prosody with dog-related content).

### Methods

#### Study site and participants

In experiment 2, 32 dogs from Redhouse Boarding Kennels in York took part (16 females and 16 males; mean age 6 years ± 3.75). Data collection for this experiment was conducted 2 years after the first experiment (2016).

#### Stimuli

For experiment 2, uncompressed WAV files were recorded from two new female experimenters (age 20 and 21). The experimenters repeated the transcripts from experiment 1 with the opposing prosody, in order to produce content-mismatched DDS and ADS. All stimuli were still directed to an appropriate live audience (e.g. adult script was produced with dog prosody to a live dog; Irish setter) and processed as described in experiment 1.

For the stimuli used in experiment 2, some dog content was repeated in ADS, and some adult content was removed in DDS. This was in order to account for differences in word rate between naturalistic DDS and ADS. These alterations are indicated in Supplementary material. The amplitude of the speech segments was again equalised, and tracks were built as in experiment 1 (see Fig. 1).

#### Acoustic analysis of stimuli

To ensure the prosody of the content-mismatched DDS and ADS for experiment 2 was convincing, we compared the acoustic properties of these stimuli with the stimuli used in experiment 1. Mean, minimum and maximum pitch (FO) was measured (Table 3) in PRAAT (version 6.0.05). Pitch settings were 75-1200 Hz and continuous segments of speech with a continuous visible pitch line were selected, and the mean, min and max pitch in the segment was extracted using the ‘get pitch’ function. Pitch modulation was calculated as maxF0-minF0. Word rate was calculated as the number of words divided by the duration from the start of the first word to the end of the last word in a stimulus.

**Table 3:** Acoustic measurements of the different types of speech produced by each experimenter

Speaker ID	Prosody	Content	Mean pitch	Pitch modulation	Word rate
Experimenter 1	DDS	Dog	598.88	240.26	172.85
	ADS	Adult	452.68	170.02	216.01
Experimenter 2	DDS	Dog	794.51	207.49	195.37
	ADS	Adult	413.47	62.97	242.40
Experimenter 3	DDS	Adult	684.58	285.92	138.97
	ADS	Dog	487.00	87.45	270.53
Experimenter 4	DDS	Adult	535.02	172.18	128.95
	ADS	Dog	472.75	83.26	278.71

Mean values from the 10- and 15-s segments are reported in each row

Generalised linear mixed models (GLMMs) were used to assess the effect of prosody (dog-directed/adult-directed); content (dog/adult) and content–prosody matching (matched (experiment 1)/mismatched (experiment 2)) on the acoustic measurements of stimuli in experiments 1 and 2. These factors were entered as fixed factors in models with (1) mean pitch and (2) pitch modulation as DVs. In order to ensure we were comparing the pitch-related measures of the same words or phrases, for mean pitch and pitch modulation, measurements of each continuous segment of speech with a continuous visible pitch line that were available in both experiments were entered into the analyses. Each speech segment was numbered and included as a random factor along with speaker identity, in order to control for repeated sampling at these two levels (Warmelink et al. 2013). For word rate, the rate of each 10- or 15-s stimulus produced by each speaker was entered into analyses, with speaker identity entered as a random factor to control for repeated sampling of each speaker. As we only had a small number of data points for this GLMM (*N* = 16), we ran three separate models, each with a single fixed factor (prosody, content or prosody–content matching) to avoid overfitting the models.

GLMMs revealed that the content-matched (experiment 1) and content-mismatched stimuli (experiment 2) did not significantly differ in pitch, pitch modulation or word rate (Tables 3, 4), indicating that the content-mismatched stimuli were produced with prosody representative of natural dog-directed and adult-directed speech. In line with previous descriptions of the prosody of DDS, the pitch was significantly higher, the pitch modulation significantly greater and word rate significantly slower for stimuli produced with dog-directed prosody compared to adult-directed prosody (Burnham et al. 1998; Ben-Aderet et al. 2017; Tables 3, 4). Content did not significantly affect pitch modulation or word rate, but dog content was significantly higher pitched than adult content (Tables 3, 4).

**Table 4:** Results of GLMMs exploring the effect of prosody, content and content–prosody matching on pitch, pitch modulation and word rate

	df	Prosody *F*(*p*)	Content *F*(*p*)	Content–prosody matching *F*(*p*)
Mean pitch	1, 328	**245.86 (**< .001)***	**13.97 (**< .001)***	0.58 (.447)
Pitch modulation	1, 328	**49.13 (**< .001)***	0.07 (.792)	0.20 (.653)
Word rate	1, 6	**34.22 (**< 001)***	3.24 (.094)	< **0.01 (.937)**

Bold value denotes a significant finding

Significant results are indicated where *** denotes *p* < 0.005

#### Design

As in experiment 1, this experiment used a within-subject design with all dogs hearing both DDS and ADS. Between-subject factors such as DDS speaker, DDS location and stimulus order were counterbalanced across trials.

#### Procedure

The procedure for this experiment was identical to that of experiment 1.

#### Interobserver Reliability

The primary observer (AB) coded 100% of videos. Two trained observers each coded 50% of the videos (*N* = 32/32 trials total). The primary observer had high agreement with both secondary coders, who also had high agreement with each other across all measurements (Spearman’s *R* > 0.90, *p* < 0.001 for all comparisons).

A third observer, who was blind to the hypotheses of the experiment, also coded 22% of the videos (*N* = 7/32 trials total) with the sound turned off so that they were unaware which speech type was heard by the dog. There was high agreement with the primary coder for looking time (*R* = 0.93, *p* < 0.001) and for proximity preference (*R* = 0.88, *p* < 0.001).

#### Statistical analysis

As above, attentive and affiliative preference was evaluated using mixed ANOVAs with the fixed within-subject factor speech prosody (DDS/ADS), between-subject factors DDS identity (e.g. experimenter 1/experimenter 2) and DDS location (right/left). All assumptions were tested and met.

### Experiment 2: results

#### Looking preference

**Fig. 5:**
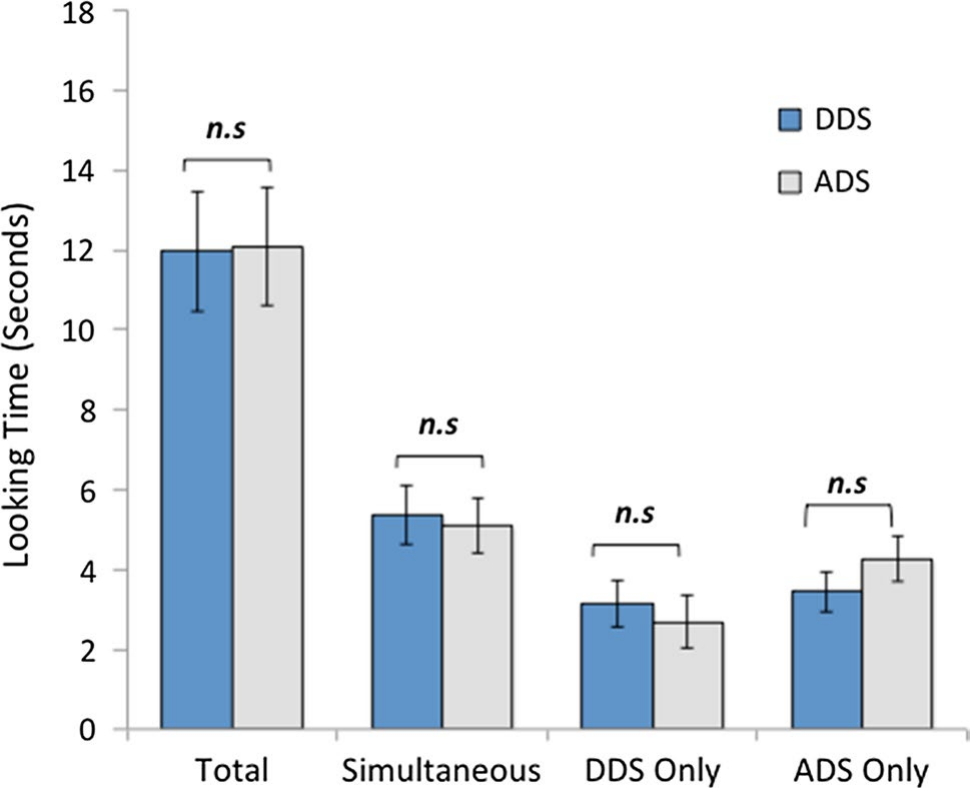
Time spent looking towards content-mismatched DDS and ADS during each phase, where error bars represent 1 standard error of the mean. n.s denotes non-significant comparisons as revealed by mixed ANOVAs (total: Table 5: other time segments: Table S4)

For content-mismatched DDS, 3 trials were removed due to equipment failure and the following analysis is based on *n* = 29. A mixed ANOVA revealed there was no significant preference for DDS when content was incongruent with prosody (Fig. 5; Table 5). During the control period, there was a main effect of identity, with dogs preferring to look towards experimenter 3 compared to experimenter 4 (Table 5). There was also an interaction of speech type and identity for total looking time. To explore the nature of the interaction between speech type and identity, four independent samples *t* tests with Bonferroni-corrected alpha (*p* < 0.0125) were conducted. Firstly, at the level of DDS, there was a significant main effect of speaker identity, with dogs preferring the speech of experimenter 3 over experimenter 4 (*t* (27) = 3.08, *p* = 0.005). However, at the level of ADS, there was no significant effect of speaker identity (*t* (27) = 0.82, *p* = 0.419). At the level of each speaker, there was no preference for the DDS of experimenter 3 compared with her ADS (*t* (27) = 0.77, *p* = 0.450), and the same was true for experimenter 4 (*t* (27) = − 1.50, *p* = 0.146).

**Table 5:** Results of between-subject ANOVA (1,25) for the control silence and a mixed ANOVA with degrees of freedom (1,25) comparing main effects and interactions for looking times towards content-mismatched DDS and ADS

	Within-subject effects *F*(*p*)				Between-subject effects *F*(*p*)		
	Speech type	Speech type *identity	Speech type * location	Speech type * identity * location	Identity	Location	Identity * location
Control silence					**4.24 (**.**048**)*	1.44 (.242)	1.02 (*.322*)
Total looking	< 0.01 (.985)	**5.75** (**.024**)*	2.03 (.167)	1.00 (.328)	2.58 (.121)	0.99 (.330)	0.34 (.560)

Bold value denotes a significant finding

Significant results are marked, where * indicates *p* < 0.050

#### Proximity preference

**Fig. 6:**
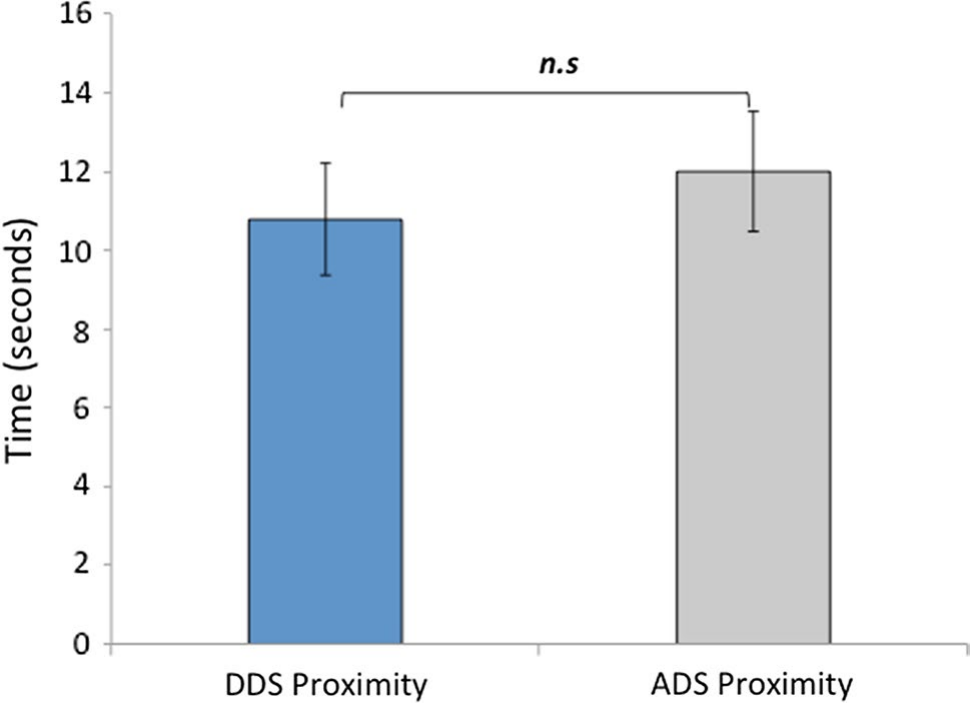
A graph to show mean time spent in proximity with each speaker (seconds), for content-mismatched DDS and ADS. Error bars represent one standard error of the mean

This analysis is based on *N* = 30 following equipment failures. For content-mismatched stimuli, dogs spent more time, on average, in proximity to the ADS location as illustrated in Fig. 6. However, a mixed ANOVA revealed that this result was non-significant (see Table 6).

**Table 6:** Results of a mixed ANOVA with degrees of freedom (1,26) comparing the time spent near DDS and ADS speakers for content-mismatched speech

	Within-subject effects *F*(*p*)				Between-subject effects *F*(*p*)		
	Speech type	Speech type *identity	Speech type * location	Speech type * identity * location	Identity	Location	Identity * location
Proximity preference	1.03 (.319)	0.85 (.365)	0.01 (.992)	0.02 (.894)	1.20 (.283)	0.59 (.448)	0.52 (.477)

To explore whether the failure to find a significant preference for either type of speech was likely due to reduced power associated with the slightly smaller sample size in experiment 2 compared to experiment 1, we considered effect sizes and conducted power analyses using G*Power (version 3.1.9.2). The preference for attending to DDS in experiment 1 was associated with a large effect size (*η*^2^ = 0.563), yet the same comparison in experiment 2 yielded a very small effect size (*η*^2^ < 0.001). An a priori power analysis for looking time in experiment 2 indicated that to find a similar effect size based on partial *η*^2^ of 0.56, with power of 0.80 and an alpha level of 0.05 for the within-subject comparison of speech type, 6 participants would have been needed, which we exceeded with our 29 participants in experiment 2. The proximity preference for the DDS speaker in experiment 1 was associated with a medium effect size (*η*^2^ < 0.156), yet the same comparison in experiment 2 yielded a small effect size (*η*^2^ = 0.038). An a priori power analysis for proximity duration in experiment 2 indicated that to find a similar effect size based on partial *η*^2^ of 0.16, with power of 0.80 and an alpha level of 0.05 for the within-subject comparison of speech type, 24 participants would have been needed, which we exceeded with our 30 participants in experiment 2. Together the effect sizes and power analysis indicate that experiment 2 had sufficient power to find differences similar to those found in experiment 1, had they existed, and therefore, we can be relatively confident in this null result.

### Discussion

The results from experiment 2 suggest that there is no significant difference in dogs’ attention or proximity preference to speakers of DDS or ADS where content and prosody did not match. This suggests that neither content, nor prosody, is solely responsible for the preference for DDS shown in experiment 1. As the same scripts were used in both experiments, this result also highlights that the preference shown in experiment 1 could not be explained by the use of specific words in the content of the original stimuli, such as ‘walk’ or ‘dog’, for example. If this were the case, we would have observed a preference for content-mismatched ADS, which not only contained the specific dog-related words used in experiment 1, but more repetitions of them (see methods).

In order to explore alternative explanations for these null results we first considered if the difficulty of producing these content-mismatched stimuli had resulted in poor examples of DDS and ADS prosody being produced. The acoustic analysis of the stimuli, however, illustrates that the content-mismatched stimuli followed the same patterns of acoustic properties as the naturalistic DDS of experiment 1. This supports the use of these stimuli and highlights that the null result found in this experiment is unlikely to be due to failures in producing authentic DDS or ADS when the content is reversed. Second, although a broadly comparable number of subjects were used in experiments 1 and 2, it is possible that the slightly smaller N available in experiment 2 (33 vs 29 Looking duration; 34 vs 30 proximity duration), left experiment 2 with slightly less power to detect differences compared to experiment 1. However, examination of effect sizes indicates that while the naturalistic speech in experiment 1 elicited large effect size (*η*^2^ = 0.563), effect sizes obtained with the reversed stimuli were extremely small (*η*^2^ < 0.001). Power analyses confirmed that we had sufficient sample sizes in experiment 2 to detect differences similar to those found in experiment 1. We are therefore confident that the null result in experiment 2 was not due to lack of power.

In experiment 2 a significant interaction between speech type and experimenter revealed that experimenter 3’s DDS was more effective at eliciting attention than experimenter 4’s DDS. This effect is likely mediated by what seemed to be an a priori preference for experimenter 1, which resulted in dogs looking significantly longer at this experimenter in the control period before any speech was produced. It is not clear whether visual or scent characteristics drove this preference, although scent seems unlikely as the preference did not remain in the post-stimulus proximity to experimenters where an attractive scent could have been actively explored. It is interesting that dogs seemed to have an immediate preference for one experimenter and this may have enhanced the efficacy of an experimenter’s dog-directed prosody. It is, however, important to note that the preferred experimenter’s DDS was still not significantly more effective in attracting dogs’ attention than her ADS. Indeed post hoc analyses of the interaction term at the level of each speaker confirmed the main findings that the different types of speech did not elicit significantly different behaviour from the dogs.

## General discussion

The results provide evidence that in an ecologically valid setting, dogs attended more towards naturalistic DDS, where prosody and content were matched, compared with ADS. We also show for the first time that dogs subsequently spend more time in proximity to an experimenter who has recently produced naturalistic DDS than one who has recently produced ADS. This novel finding suggests that DDS may fulfil a dual function of improving attention and increasing social bonding. This fits with the current understanding of infant research, which suggests not only that IDS serves to facilitate language acquisition, but that it is also crucial for developing meaningful social relationships with caregivers.

The second experiment was designed to investigate whether prosody or content alone was driving this preference for naturalistic DDS; however, when content and prosody were mismatched, we found there was no difference in the amount of time spent in proximity to the experimenters and there was no significant attentive preference for DDS or ADS in any part of the trial, or across the session as a whole. This suggests that neither content, nor prosody alone was driving the preference observed in experiment 1. Instead, it is clear that both content and prosody matter to dogs. Future research should aim to disentangle whether dog-related prosody and content independently affect dog behaviour, or whether they have to be combined congruently in order to affect dog preferences. This study is unable to distinguish between these possibilities; however, the results from Ben-Aderet et al. (2017), who found that adult dogs did not prefer dog-relevant content produced with dog-directed prosody over adult-directed prosody, indicate that it may be the congruent combination of dog-directed content and prosody that underpins the preference for naturalistic DDS. Further experiments, with large sample sizes, which manipulate both prosody and content independently, are required to understand this relationship more fully.

Interestingly, Ben-Aderet et al. did find a significant preference for DDS prosody in puppies, showing that puppies are more sensitive to prosodic differences compared to adult dogs. Puppies may be more sensitive to acoustic differences than adult dogs in the same way that human babies are most sensitive to IDS early in life (Newman and Hussain 2006). Puppies also have less experience of human language and time to form associations between specific words and positive experience (e.g. walk) and thus should be less sensitive to content. Therefore, while puppies may rely wholly on prosodic information, adult dogs seem to take both content and prosody into account, and only when these two things are relevant to them, they do display a behavioural preference. While preference for dog-related content needs experience of human interaction to develop, the origins of the preference for dog-directed prosody are less clear: they may be routed in an innate preference for higher pitched, tonal sounds, the domestication process or be a product of early learning environment. If preferences for DDS prosody are based on preferences for high pithed tonal sound, which across mammalian species is associated with affiliation and submission rather than aggression (Morton 1977), then other mammalian species should show a preference for DDS over ADS. Future research could test this possibility. Alternatively, preference for DDS prosody may have arisen through various routes during the domestication process. Firstly, early in the domestication process, DDS may have provided dogs with a reliable cue that indicates safe social partners at a time when joining human groups may have been dangerous, and identifying those who would not be hostile would have been important for a dog’s survival. Secondly, as dogs are able to engage with humans in joint attention (Miklósi et al. 2003) and can cooperate to achieve goal-directed actions (Range and Virányi 2014), it is possible that humans selected dogs for characteristics that promoted social communication during domestication, including attentive and affiliative preference for DDS. It is, however, also possible that dogs kept as pets are conditioned over their individual lifetimes to respond positively to DDS as this type of speech is often paired with positive events (e.g. food treat, toy, walk or affection). Although Ben-Aderet et al. found a clear preference for DDS in young dogs (2–5 months), it is possible that such associations could be formed in that time. Future research with young puppies raised with extremely minimal human contact would enable us to test whether environmental input is needed to shape this preference or whether it is an innate preference, as it seems to be in human infants (Cooper and Aslin 1990).

Although the use of real people to deliver the speech to the dogs increased the ecological validity of our experimental set-up, it did have potential drawbacks. First, the importance of providing speech from each experimenter (exact match with characteristics including gender, height and size) to ensure it was physically congruous meant that the same stimuli were heard by multiple dogs. Although acoustic analysis confirmed the structure of these stimuli were representative of DDS and ADS reported in other studies, it is unclear whether these findings would generalise to a wider sample of DDS and our findings suggest that there may be individual variation in the efficacy of DDS. Thus, further studies without pseudoreplication at the level of the stimulus are required to confirm the generalisability of our findings. Differential a priori interest in the experimenters, as we found in experiment 2, is a further complication associated with the use of live models in these experiments, which highlights the need for rigorous counterbalancing and a control period where such a priori biases can be measured. In addition, our results illustrate the interesting possibility that a priori preferences for individuals may influence the effectiveness of and sensitivity to other cues including speech register.

In conclusion, the results from this study support the hypothesis that dogs pay more attention to naturalistic DDS than to ADS. It also revealed that dogs spent more time near someone who had just produced DDS rather than ADS, indicating for the first time that DDS may not just modulate attentive behaviour, but also play a role in the development of affiliative preferences. This preference for naturalistic DDS was not driven by preference for dog-directed content or prosody alone, as no attentive or affiliative preferences were shown when dogs were presented with content- and prosody-mismatched stimuli. This study concludes that naturalistic DDS elicits more attention from dogs than ADS and has the potential to strengthen the affiliative bond a human has with a dog.

## Electronic supplementary material

Below is the link to the electronic supplementary material.

Supplementary material 1 (DOCX 137 kb)
