# Supplementary material for: ‘Who’s a good boy?!’ Dogs prefer naturalistic dog-directed speech
Source: Anim Cogn. 2018 Mar 2;21(3):353–64. doi: 10.1007/s10071-018-1172-4 (PMC5908831; doi:10.1007/s10071-018-1172-4)
Supplement: Supplementary file 1 — Supplementary material 1 (DOCX 137 kb) [file 10071_2018_1172_MOESM1_ESM.docx]

Supplementary Material

Supplementary Methods: Stimuli

**Parts of scripts underlined represent segments that were removed, and in bold indicate segments of the speech that were repeated in experiment 2 to account for differences in word rate between DDS and ADS**

Content-Matched DDS

Experimenter 1: *‘Oh you’re such a good dog… Yes you are… Come here… Come here… You’re such a good dog yes you are… Good dog… You’re such a good dog yeah… You’re such a good dog.’ (10 Seconds).*

*‘Come on come here… Good dog… Come here good dog… You’re such a good dog… yes you are… come here… come here… good dog… yes you are… come on … come here… yeah come on… good dog… good dog.’ (15 Seconds).*

Experimenter 2: *‘Oh you’re such a good dog… you gonna come here?... you gonna come? Come on then… come on… let’s go out… lets go for a walk… oh you’re such a good dog… yes you are.’ (10 Seconds).*

*‘Oh good dog… you gonna come here? Come on then… come on… let’s go out for a walk… shall we go for a walk… come on then… come here… lets go for a walk… oh you’re such a good dog, yes you are… you’re such a good dog.’ (15 seconds).*

Content-Matched ADS

Experimenter 1: *‘So I had a massive meltdown with my lit survey last night… I found out that the topic I was doing didn’t have enough literature on… or* *I couldn’t get access to the papers… so I had to email my supervisor’ (10 Seconds).*

*‘I went to the gym yesterday and I joined this new class… but it… we had to do like… 15 minutes of aerobic exercise followed by all these weights and squats and crunches and things… um… and then I made the mistake of going into the pool afterwards and did a couple of lengths but… today I’m really feeling it.’ (15 Seconds).*

Experimenter 2: *‘I went to the cinema last night and saw a really good film, it was really funny I really think you should go and see it… it was a comedy about this … err… this girl who was getting married.’ (10 Seconds).*

*‘I went to the gym yesterday and joined this new fitness class. It was really, really hard. You had to do, like, 15 minutes of aerobic exercise and then sit-ups and weights… ugh, it was just so difficult… and then afterwards I thought I’d cool off in the pool… but I think that was a mistake because I’m really feeling it this morning.’ (15 Seconds).*

Content-Mismatched DDS

Experimenter 3: *‘So (gasp)* I had a massive meltdown with my lit survey last night… (gasp) I couldn’t get access to the papers (gasp) and I had to email my supervisor.’ (10 Seconds).*

*‘(Gasp) I went to the gym yesterday and I joined this new class... we had to do… 15 minutes of aerobic exercise… followed by all of these weights (gasp) and squats (gasp) and crunches and things.’ (15 Seconds).*

Experimenter 4: *‘(Gasp) I went to the cinema last night… (gasp) and saw this really good film… It was really funny… and I think you should go and see it (gasp).’ (10 Seconds).*

*‘(Gasp) I went to the gym yesterday and joined this new fitness class. It was really, really hard, you had to do like, (gasp) fifteen minutes of aerobic exercise, and then… I had to cool off in the pool but that was a mistake.’ (15 Seconds).*

Content-Mismatched ADS

**Experimenter 3:** *‘Oh you’re such a good dog… yes you are… come here... come here… you’re such a good dog, yes you are… You’re such a good dog, yeah… you’re such a good dog.* ***Oh you gonna come here? You gonna come? Come on then, come on, let’s go out****.’ (10 Seconds).*

*“Come on, come here… good dog, come here… good dog… you’re such a good dog… yes you are… come here… come here… good dog… yes you are… come on, come here… yeah come on… good dog… good dog.* ***Oh good dog, you gonna come here? Come on then, come on, let’s go for a walk. Shall we go for a walk? Come here, let’s go for a walk****.” (15 Seconds)*

Experimenter 4: *‘Oh good dog, you gonna come here? Come on then, Come on... Let’s go for a walk, shall we go for a walk? Come on then, come here... Let’s go for a walk, oh you’re such a good dog, yes you are, you’re such a good dog…* ***come here, good dog****.’ (10 Seconds).*

*‘Oh good dog, you gonna come here? Come on then, come here, let’s go for a walk, shall we go for a walk? Come on then, come on, come here… Let’s go for a walk, oh you’re such a good dog, yes you are… You’re such a good dog,* ***come here, come here, oh good dog come here you’re such a good dog yes you are****.’ (15 Seconds).*

Supplementary Methods: Participants

Table S1. List of Dogs in Experiment 1: Content-Matched Stimuli. DDS Preference is also indicated, calculated as total time spent looking towards the DDS speaker minus total time spent looking towards the ADS speaker. Therefore positive values indicate a preference for DDS, while negative values indicate a preference for ADS.

| **Dog Name** | **Gender** | **Breed** | **Year of Birth** | **DDS Preference** |
| --- | --- | --- | --- | --- |
| Harvey | Male | Collie | 2010 | 13 |
| ShiPoo | Female | Shih tzu -Poodle Cross | 2013 | 27 |
| Tilly | Female | Cocker Spaniel | 2013 | 2 |
| Toby | Male | Cocker Spaniel | 2008 | 5.5 |
| Teddy | Male | Chow Chow | 2002 | 0 |
| Wilson | Male | Staffordshire Bull Terrier | 2006 | 5 |
| Holly | Female | Collie | 2004 | 5 |
| Kaiser | Male | Schnauzer | 2008 |  |
| Millie | Female | Cairn Terrier | 2003 | 21 |
| Nellie | Female | Fox Terrier | 2009 | 9 |
| Roxy | Female | Bichon Frise | 2009 | 18.5 |
| Tess | Female | Lakeland Terrier Mix | 2007 | 7 |
| Bailey | Male | Cockerpoo | 2012 | 5.5 |
| Rua | Male | King Charles Spaniel | 2008 |  |
| Buster | Male | King Charles Spaniel | 2009 | 17 |
| Doug | Male | Cocker Spaniel | 2010 | 17 |
| Doyle | Male | Jack Russell-Staffordshire Bull Terrier Cross | 2008 | 0 |
| Tom | Male | Lab-Collie Cross | 2001 | 3.5 |
| Lucy | Female | Labrador | 2012 | 2 |
| Dibley | Male | Cairn Terrier | 2003 | 7 |
| Fin | Male | Border Collie | 2003 | 12 |
| Lulu | Female | Bichon Frise | 2012 | -2 |
| Mertie | Female | Miniature Schnauzer | 2003 | 5.5 |
| Willow | Female | Springer Spaniel Mix | 2012 |  |
| Sparkle | Female | West Highland Terrier | 2003 |  |
| Penny | Female | Collie | 2006 | 5 |
| Poppy | Female | Cockapoo | 2009 | 21 |
| Arnie | Male | Staffordshire Bull Terrier | 2010 | 4.5 |
| Billie | Male | Shih tzu | 2012 | 7 |
| Stella | Female | Staffordshire Bull Terrier Mix | 2013 | 6 |
| Ted | Male | Nova Scotian Duck Tolling Retriever | 2013 | 5 |
| Alfie | Male | Boarder Terrier | 2002 | 1.5 |
| Lewis | Male | Singapore Street dog | 2008 | -6.5 |
| Barnaby | Male | Singapore Street dog | 2010 | 19 |
| Ben | Male | Labrador - lurcher cross | 2011 | 4 |
| Millie2 | Female | Jack Russell | 2013 | 13 |
| Pip | Female | Jack Russell | 2013 | 7 |

Table S2. List of Dogs in Experiment 2: Content-mismatched stimuli. DDS Preference is also indicated, calculated as the total time spent looking towards the DDS speaker minus the total time spent looking towards the ADS speaker. Therefore positive values indicate a preference for DDS, while negative values indicate a preference for ADS.

| **Dog Name** | **Gender** | **Breed** | **Year of Birth** | **DDS Preference** |
| --- | --- | --- | --- | --- |
| Reubin | Male | Lhasa Apso-Poodle Cross | 2012 | 2 |
| Dinky | Female | Jack Russel | 2006 | 0 |
| Alfie | Male | Irish Setter | 2014 | -1.5 |
| Skye | Male | German Shepherd-Husky Cross | 2013 | -31 |
| Loki | Male | Staffordshire Bull Terrier | 2015 | -10.5 |
| Harry | Male | Yorkshire Terrier | 2007 | 4 |
| Buddy | Male | Staffordshire Bull Terrier | unknown | -3.5 |
| Ali | Male | Staffordshire Bull Terrier | 2008 | 3 |
| Albert | Male | Yorkshire Terrier | 2007 | 16.5 |
| Tip | Female | Border Collie | 2002 | -0.5 |
| Samson | Male | Labrador | 2014 | -1 |
| Otto | Male | Cockapoo | 2015 | -4 |
| Nutmeg | Male | Labrador | unknown | -7 |
| Jazz | Female | Border Terrier | 2008 | 2.5 |
| Jake | Male | Mixed Breed | unknown | -4 |
| Alfie2 | Male | Beagle | unknown | -9 |
| Vincent | Male | Pug | 2010 | 21.5 |
| Sam | Male | Jack Russel-Patterdale Terrier Cross | 2009 | -22.5 |
| Rico | Male | Chihuaha | 2015 | 3.5 |
| Phoebe | Female | Pug | 2011 | -7.5 |
| Ella | Female | Cocker Spaniel | 2013 | 14 |
| Sally | Female | Pug-Cavalier King Charles Spaniel Cross | 2008 | -4.5 |
| Lilly | Female | Labrador | 2006 | -9 |
| Hugo | Male | Shitzhu-Lhasa Apso Cross | 2014 | 0 |
| Chuck | Male | Cavalier King Charles Spaniel | 2013 | -11.5 |
| Bella | Female | Miniture Shnauser | 2015 | 10 |
| Satu | Female | Labrador | 2013 | 24 |
| Mitsy | Female | Yorkshire Terrier Mix | 2006 | 2 |
| Charlie | Female | Chihuaha | unknown | 21 |

Supplementary Results

Table S3. Results of a between subjects ANOVA (1,29) for looking time during the control period and a Mixed ANOVA with Degrees of Freedom (1,29) comparing main effects and interactions for looking times towards content-matched DDS and ADS. Significant results are marked, where * = *p*<0.050 and ** = *p*< 0.005 for total looking time and control silence. Bonferoni corrected alpha was employed for individual segments of the stimulus (*p=*.0125). No significant effects or interactions were found at this level.

|  | **Within Subject Effects F(*p*)** | | | | **Between Subject Effects F(*p*)** | | |
| --- | --- | --- | --- | --- | --- | --- | --- |
|  | Speech Type | Speech Type *Identity | Speech Type * Location | Speech Type * Identity * Location | Identity | Location | Identity * Location |
| **Control Silence** |  |  |  |  | 0.38 *(.543)* | 0.59 (*.448*) | 0.85 (*.364)* |
| **Simultaneous** | **30.12 *(<.001)***** | 1.43 *(.241)* | 2.76 *(.107)* | <0.01 *(959)* | 0.70 *(.408)* | 0.08 *(.785)* | 0.17 *(.685)* |
| **DDS Only** | **48.46 *(<.001)***** | 0.04 *(.841)* | 0.35 *(.562)* | 0.00 *(.961)* | 2.83 *(.103)* | 4.54 *(.042)* | 0.36 *(.551)* |
| **ADS Only** | 3.81 *(.061)* | *2.29 (.141)* | 1.92 *(.176)* | 0.03 *(.855)* | 0.37 *(.548)* | 0.16 *(.692)* | 0.26 *(.614)* |
| **Total Looking** | **40.51 *(<.001)****** | 0.15 *(.704)* | 1.61 *(.215)* | 0.24 *(.627)* | 0.20 *(.656)* | 1.37 *(.251)* | 0.43 *(.517)* |

Table S4. Results of Mixed ANOVA with Degrees of Freedom (1,25) comparing main effects and interactions for looking times towards content-mismatched DDS and ADS. Significant results are marked, where * = *p*<.050 and ** = *p*< .005 for control silence and total looking time. Bonferoni corrected alpha was employed for individual segments of the stimulus (*p=.*0125). No significant effects or interactions were found at this level.

|  | **Within Subject Effects F(*p*)** | | | | **Between Subject Effects F(*p*)** | | | | | |
| --- | --- | --- | --- | --- | --- | --- | --- | --- | --- | --- |
|  | Speech Type | Speech Type *Identity | Speech Type * Location | Speech Type * Identity * Location | | Identity | Location | Identity * Location | |  |
| **Control Silence** |  |  |  |  | | **4.24** **(*.048****)** | 1.44 (*.242)* | | 1.02 (*.322*) | |
| **Simultaneous** | 0.18 (*.678*) | 2.15 (*.155*) | 2.51 (*.126*) | 2.53 (*.125*) | | 0.53 (*.474*) | 0.23 (*.635*) | | 0.02 (*.899*) | |
| **DDS Only** | 0.15 (*.702*) | 6.33 (*.019*) | 0.10 (*.756*) | 0.09 (*.765*) | | 1.80 (*.192*) | 0.93 (*.345*) | | 0.24 (*.628*) | |
| **ADS Only** | 1.02 (*.322*) | 1.77 (*.195*) | 1.34 (*.257*) | 0.56 (*.463*) | | 3.38 (*.078*) | 0.90 (*.351*) | | 1.30 (*.266*) | |
| **Total Looking** | <0.01 (*.985*) | **5.75** (***.024***)* | 2.03 (*.167*) | 1.00 (*.328*) | | 2.58 (*.121*) | 0.99 (*.330*) | | 0.34 (*.560*) | |
